# CD4^+^ T Cell Subset Differentiation and Avidity Setpoint Are Dictated by the Interplay of Cytokine and Antigen Mediated Signals

**DOI:** 10.1371/journal.pone.0100175

**Published:** 2014-06-18

**Authors:** Erin K. Shiner, Beth C. Holbrook, Martha A. Alexander-Miller

**Affiliations:** 1 Department of Microbiology and Immunology, Section on Rheumatology and Immunology, Wake Forest School of Medicine, Winston-Salem, North Carolina, United States of America; 2 Department of Internal Medicine, Section on Rheumatology and Immunology, Wake Forest School of Medicine, Winston-Salem, North Carolina, United States of America; University of Iowa, United States of America

## Abstract

CD4^+^ T cell differentiation has been shown to be regulated by the cytokine milieu present during activation as well as peptide MHC levels. However, the extent to which these two important regulatory signals work in concert to shape CD4^+^ T cell function has not been investigated. Using a murine OT-II transgenic TCR model of *in vitro* differentiation, we demonstrate that the ability of CD4^+^ T cells to commit to a distinct lineage, i.e. Th1 vs. Th2 vs. Th17, is restricted by the amount of peptide antigen present in the stimulating environment. In addition, whether cells succumb to inhibitory effects associated with high dose antigen is dependent on the array of cytokine signals encountered. Specifically, stimulation with high dose antigen in Th1 or Th17 conditions promoted efficient generation of functional cells, while Th2 polarizing conditions did not. Finally, we found that the peptide sensitivity of an effector cell was determined by the combined actions of cytokine and peptide level, with Th1 cells exhibiting the highest avidity, followed by Th17 and Th2 cells. Together, these data show that the interplay of antigen and cytokine signals shape both the differentiation fate and avidity setpoint of CD4^+^ T cells.

## Introduction

Following activation, naïve CD4^+^ T cells will undergo a program of differentiation that results in the ability to produce a defined set of cytokines [1]. The nature of cytokines produced by the differentiated CD4^+^ T cells identifies them as one of a number of distinct subsets that include, but are not limited to Th1, Th2 and Th17. The fate choice of these cells has profound implications for their function in vivo. Th1 cells are defined by the production of high levels of IFNγ and play a critical role in the clearance of intracellular pathogens. While this has been thought to occur primarily through their support of CD8^+^ T cell and B cell activation/function, it is increasingly clear that Th1 cells can play a direct role in pathogen clearance through a variety of mechanisms including cytolysis, IFNγ production, and enhancement of innate inflammatory cytokines and chemokines [2]–[4]. Th2 cells produce a number of cytokines including IL-4 and IL-5. These cells support B cell production of high affinity antibody which is efficient in the clearance of extracellular parasites. The more recently described Th17 subset is a key mediator of the inflammatory response. Among their functional attributes is the recruitment of neutrophils that are necessary for the clearance of extracellular bacterial and fungal infections [5].

CD4^+^ T cell differentiation is regulated by cytokine signals present in the environment during their activation and expansion [1]. IL-12 together with IFNγ induce Th1 cell development, while IL-4 drives differentiation into Th2 effectors. Th17 cells are generated as a result of signals from low dose TGFβ in combination with inflammatory cytokines such as IL-6 or IL-21. Cytokine mediated differentiation is controlled by a defined signal transducer and activator of transcription (STAT) and a master regulatory transcription factor (for review see [1]). For Th1 these are STAT4 and T-bet, for Th2 STAT-5 and GATA-3, and for Th17 STAT-3 and RORγt (for review see [1]).

In addition to cytokines, antigen dose is recognized as a regulator of CD4^+^ T cell subset differentiation [6]–[11]. The vast majority of the studies in this area have focused on Th1 vs. Th2 differentiation. While the conclusions from the studies of antigen dose driven differentiation may appear to disagree in some cases, direct comparison is often complicated by the limited dose ranges chosen for evaluation [6], [7], [9]–[11]. However, in total, the data generated have led to the proposal of a biphasic model for antigen mediated differentiation where, in the absence of added differentiating cytokines, limiting or high doses of peptide promote Th2 differentiation whereas intermediate doses skew towards Th1 development [6].

The regulatory effect of peptide/MHC (pMHC) level has been most highly studied in the context of CD8^+^ T cell differentiation, where it has been identified as an important regulator of T cell avidity. Functional avidity is defined by the amount of antigenic peptide required to elicit T cell activation or effector function, with high avidity cells exhibiting greatly increased sensitivity to pMHC [12], [13]. This property is a critical attribute of effector cells. Multiple studies have shown that higher functional avidity is associated with superior in vivo efficacy for pathogen clearance [12], [14]–[20].

Our previous studies of CD8^+^ T cells demonstrated that avidity is a plastic property that can be modulated, leading to alteration of the effector function of T cells [21], [22]. Whether this is the case for CD4^+^ T cells is less clear. There is some evidence that the level of peptide used for stimulation can impact avidity. Rees *et al.* showed that multiple exposures to low dose antigen resulted in the generation of CD4^+^ T cells with high affinity for pMHC [23]. Further, an inverse correlation has been reported between CD4^+^ T cell avidity and the level of the peptide used to stimulate the cells [24].

While antigen dose and cytokines have been studied in isolation, it is unclear how the interplay of these two signals contributes to the function of CD4^+^ T cells with regard to differentiation and peptide sensitivity. To address this question, we examined the combined effect of changes in cytokine environment and peptide level, with the hypothesis that cytokine and antigen dose exert interdependent effects on the differentiation fate and peptide sensitivity in CD4^+^ T cells. Our results establish the critical regulatory role of the interplay of these two signals showing that the differentiation fate and avidity setpoint are a result of the combined actions of cytokine and TCR signals.

## Materials and Methods

### Ethics Statement

All animal work was conducted according to relevant national and international guidelines and was approved by the Animal Care and Use Committee at Wake Forest University School of Medicine.

### Mice

C57BL/6 and B6-LY5.2/Cr (expressing Ly5.1) congenic mice were purchased from the Frederick Cancer Research Development Center (Frederick, MD). OT-II TCR transgenic RAG2^−/−^ mice, which bear a TCR that recognizes a peptide from ovalbumin (OVA_323–339_) presented by I-A^b^ [25], [26] were obtained from Taconic (Germantown, NY).

### Cell Line Generation

For generation of effector cell lines, 5×10^5^ OT-II splenocytes were stimulated weekly with 5×10^6^ C57BL/6 splenocytes (2000 rad irradiated) in the presence of 10^−5^M, 10^−7^M or 10^−9^M OVA_323–339_ peptide. Cultures were supplemented with the following to promote differentiation: Th1: hIL-2 (10 U/ml)+ mIL-12 (10ng/ml) and neutralizing anti-IL-4 antibody (1 µg/ml); Th2: hIL-2 (25 U/ml)+mIL-4 (10ng/ml) and neutralizing anti-IL-12 (1 µg/ml)+anti-IFNγ (1 µg/ml) antibody; Th17: hIL-2 (10 U/ml)+mIL-6 (20ng/ml)+hTGF-β (5ng/ml) and neutralizing anti-IL-4, anti-IL-12 and anti-IFNγ antibody (all at 1 µg/ml) [27]–[30]. Cytokines were purchased from Peprotech and anti-cytokine antibodies from Biolegend. It has been shown that aryl hydrocarbon receptor agonists, which are rich in IMDM medium, are vital for Th17 polarization in vitro [29]. Thus, Th17 polarized cultures were grown in IMDM medium containing L-glutamine, HEPES, 10% FCS, 100 U/ml penicillin, 100ug/ml streptomycin, 50uM β-ME, 1% NEAA and 1mM sodium pyruvate. Th1 cells were also grown in the IMDM medium to maintain consistency in culture conditions. However, in our hands, Th2 cells differentiated poorly on IMDM medium. Therefore, they were grown in RPMI 1640 medium supplemented as above. Cultures were maintained in 24-well plates.

### Measurement of CD25 and CD69 Following Activation

5×10^5^ OT-II cells were co-cultured with 5×10^6^ irradiated CD45.1^+^ splenocytes (CB6-LY5.2/Cr), peptide (10^−5^, 10^−7^, or 10^−9^M), and cytokines for differentiation of Th1, Th2, or Th17 cells (as described above). At 0, 24, 48, or 72h, cells were removed from culture and stained with antibodies to CD4, CD45.2, CD69, and CD25. The percent of cells positive for CD25 or CD69 as well as the level of expression for each molecule within the CD4^+^CD45.2^+^ (OT-II) population was quantified.

### Analysis of T cell Death

5×10^5^ OT-II cells were co-cultured with 5×10^6^ irradiated CD45.1^+^ splenocytes (CB6-LY5.2/Cr), peptide (10^−5^, 10^−7^, or 10^−9^M), and cytokines for differentiation of Th1, Th2, or Th17 cells (as described above). At 72h, cells were removed from culture and stained with antibodies to CD4 and CD45.2. 7-AAD staining was performed using the buffer provided as per the manufacturer’s instructions (BD Biosciences).

### Measurement of T cell Function

Assessment of function was performed by co-culturing 2×10^5^ effector cells (d7 post stimulation) with 5×10^5^ C57BL/6 splenocytes in the presence of peptide (100 µM), PMA (50 ng/ml)+ionomycin (500 ng/ml), or immobilized anti-CD3 antibody (plate coated overnight with 50 µg/ml). In order to facilitate exclusion of stimulator cells from the OT-II cells, one of two approaches were used. First, when available, congenic mice that express the CD45.1 antigen were used and the OT-II cells were separated from the stimulator cells with flow cytometric analysis using anti-CD45.2 antibody. Alternatively, freshly harvested splenocytes were labeled with 25nM CFSE and the OT-II cells identified with flow cytometric analysis as the CFSE negative population. GolgiStop or GolgiPlug (BD Biosciences) was included as recommended by the manufacturer for the particular cytokine measured. For determination of functional avidity effector cells were stimulated with titrated concentrations of OVA_323–339_ peptide (10^−4^M–10^−10^M). Following a five hour stimulation, cells were stained with anti-CD4 and anti-CD45.2 antibodies. Cells were permeabilized with Cytofix/Cytoperm (BD Biosciences) and stained with anti-IFNγ and anti-IL-17 or with anti-IL-4 antibody. All antibodies were purchased from Biolegend or BD Biosciences. Cells were acquired using a FacsCanto II (BD Biosciences) and analyzed with either FlowJo (Tree Star Inc) or CellQuest software (BD Biosciences). For avidity determination, the half-maximal cytokine production, i.e. the amount of peptide required to achieve cytokine production in 50% of the cells capable of responding, was calculated by Prism (Graphpad Software Inc) from the dose-response curve data following nonlinear regression curve fitting (log agonist versus normalized response variable slope).

### Proliferation Assay

OT-II splenocytes (1×10^7^) were labeled in 2ml of PBS containing 5 µM CFSE (Invitrogen) for 15 min at room temperature. The reaction was quenched by adding FCS to 10% followed by washing with PBS. 5×10^5^ CFSE labeled OT-II cells were cultured with 5×10^6^ irradiated splenocytes in the presence of peptide and Th1, Th2 or Th17 polarizing cytokines per well of a 24-well plate. At 72h, CFSE in OT-II cells stimulated under each condition was determined.

### Statistical Analysis

Statistical analyses were performed using a student t-test, one-way ANOVA with a post-hoc Tukey test or two way ANOVA with a post-hoc Bonferroni test as appropriate (Prism Graphpad Software Inc.). A p value ≤0.05 was considered significant.

## Results

### Cytokine Restricts the Antigen Dose Range on which Cells can Survive and Differentiate

It is well established that both antigen dose and cytokines can impact CD4^+^ T cell differentiation [1], [6]–[11]. However, our understanding of how the concurrent presence of these two signals modulates function and survival is highly limited. To address this question, OT-II TCR transgenic cells, which express a TCR specific for the OVA_323–339_ peptide presented by I-A^b^ [25], were stimulated with a low (10^−9^M), intermediate (10^−7^M), or high (10^−5^M) concentration of OVA_323–339_ peptide. Preliminary studies determined that these concentrations spanned the range of OT-II responsiveness. Use of the OT-II transgenic TCR mouse model eliminated baseline variability in the TCR affinity for cognate antigen/MHC complex. Cultures were supplemented with the following to promote differentiation: Th1: hIL-2+ mIL-12 and neutralizing anti-IL-4 antibody; Th2: hIL-2+ mIL-4 and neutralizing anti-IL-12+ anti-IFNγ antibody; Th17: hIL-2+ mIL-6+ hTGF-β and neutralizing anti-IL-4, anti-IL-12 and anti-IFNγ antibody [27]–[30]. We determined that five weekly rounds of stimulation produced the maximum level of differentiation in the various cell lines and thus data shown are from that timepoint. Importantly, however, the results at this timepoint reflect the differentiation patterns present in the cultures following each round of stimulation, i.e. while the percent of functional cells may increase with multiple stimulations, the cytokines they produced were similar across all stimulations (data not shown).

**Figure 1:**
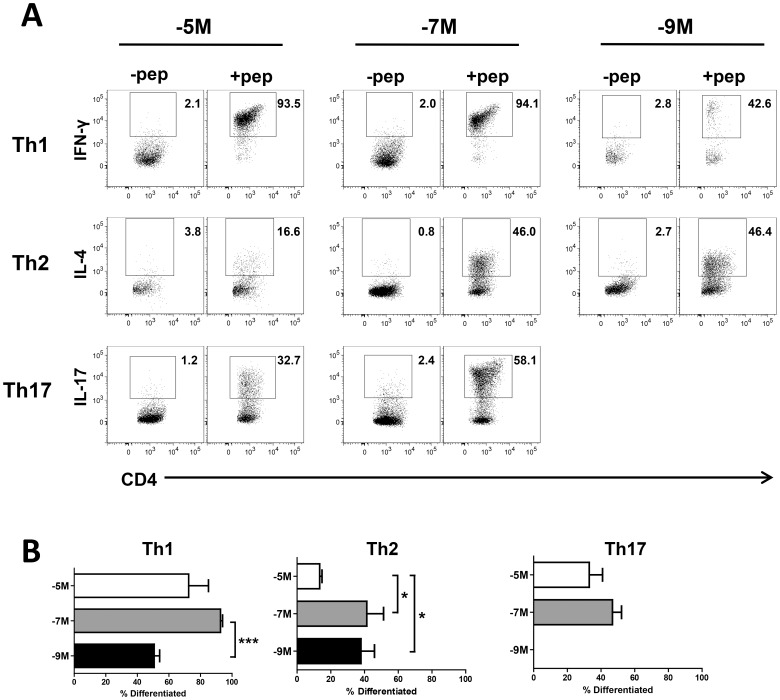
The differentiation of CD4^+^ T cells is affected by both cytokine and antigen dose. OT-II lines were generated by repeated weekly stimulation in the presence of cytokine that polarized the cells toward differentiation into Th1, Th2 or Th17 cells together with high (10^−5^M), intermediate (10^−7^M) or low (10^−9^M) concentrations of peptide. Established OT-II cell lines were assayed for cytokine production following stimulation with OVA_323–339_ peptide (10^−4^M). The differentiation state of the cell lines was determined by measuring the production of IFN-γ (Th1), IL-4 (Th2) or IL-17 (Th17). A. Representative dotplots of cytokine production for each cell line are shown. Data shown are gated on CD4^+^CD45.2^+^ cells. B. The percentage of cells that produce cytokine following peptide stimulation is shown for each stimulation condition. Th17 cells did not survive on low dose peptide. Data are the average of independently generated lines: −5M Th1 (n = 3), −7M Th1 (n = 3), −9M Th1 (n = 4), −5M Th2 (n = 3), −7M Th2 (n = 3), −9M Th2 (n = 3), −5M Th17 (n = 4), −7M Th17 (n = 3), and −9M Th17 (n = 2). * p≤0.05, *** p≤0.0005.

The differentiation state of cells within each culture condition was determined by stimulating with OVA_323–339_ peptide. IFNγ was used as an indicator of Th1 differentiation, IL-4 of Th2 differentiation and IL-17 of Th17 differentiation (Fig. 1). Th1 conditions appeared least restrictive as differentiation was observed at all concentrations of peptide used for stimulation. However, the lowest peptide concentration resulted in acquisition of cytokine producing capability in a lower percentage of cells compared to the intermediate dose. In contrast, Th2 cells differentiated efficiently at intermediate and low doses of peptide, with only a very limited percentage of functional cells generated following stimulation with10^−5^M peptide (Fig. 1). In the cells cultured using Th17 conditions, yet a third pattern of responsiveness was observed. Th17 cells were generated on high and intermediate peptide concentrations, but the low peptide dose could not support cell survival (Fig. 1). These data reveal an unexpected peptide dose restriction for the generation of functional T cells that is determined by the array of cytokines present in the environment.

**Figure 2:**
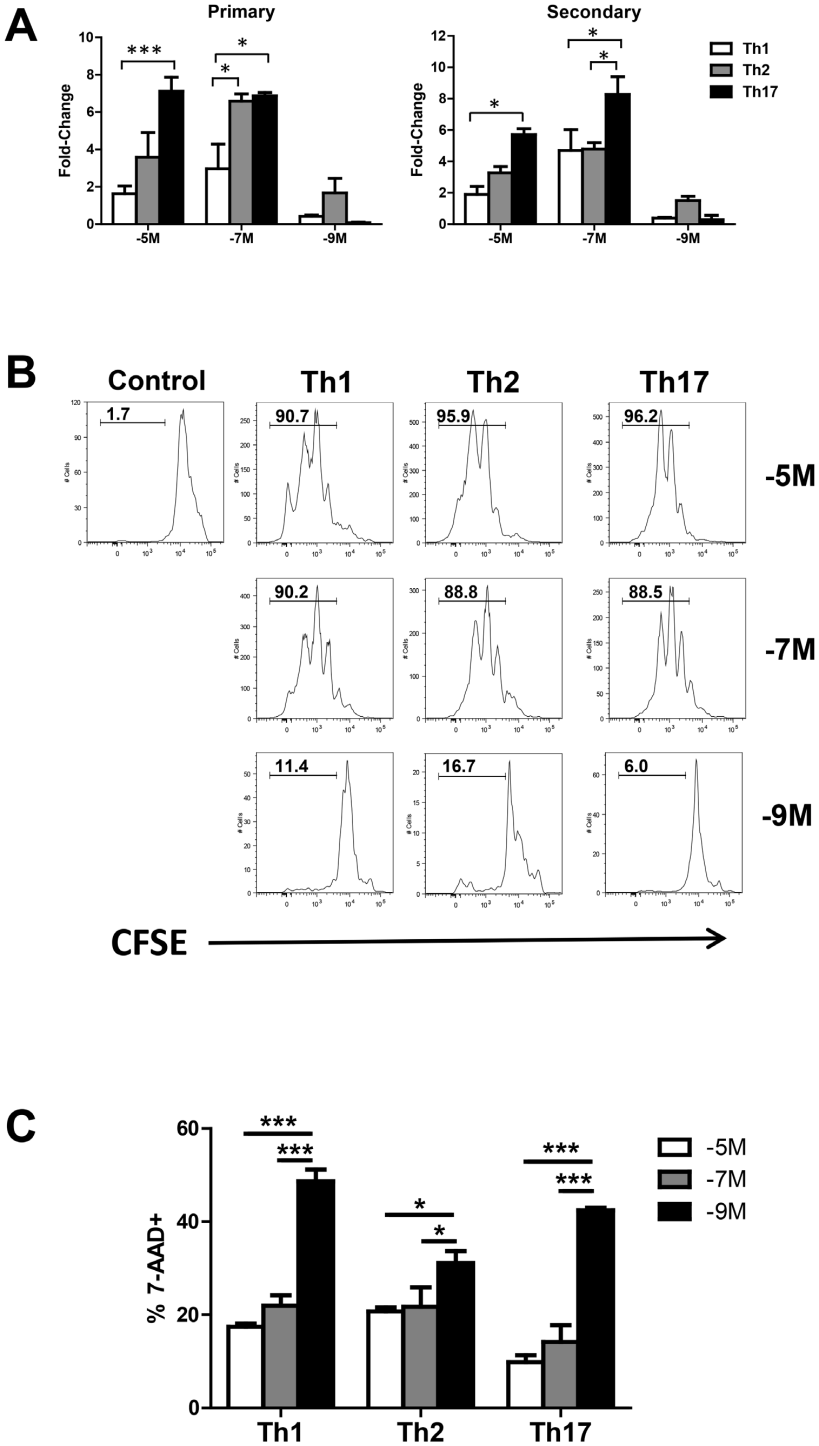
Proliferation and survival are dependent on the combined effects of peptide and cytokine environment. OT-II splenocytes from naïve mice were stimulated with a high, intermediate or low concentration of peptide in the presence of Th1, Th2, or Th17 differentiating conditions. A. Cell recovery for each condition was assessed on d7 post-primary and d7 post-secondary stimulation. Data are presented as recovery on d7 post stimulation divided by input cell number at initiation of each stimulation culture. All cultures began with the same number of input cells. Averaged data are from 2–4 independent experiments, as detailed in Figure 1. B. CFSE-labeled naïve OT-II cells were cultured for 72h in the presence of differentiating cytokines and peptide. OT-II cells cultured in the absence of peptide are shown as a control. Values shown in the histograms are the percent of cells in the divided gate. C. At 72h following initiation of primary culture, cells were incubated with 7-AAD as a measure of cell death. OT-II cells were identified by CD4 and CD45.2 positivity. All data are representative of three independent experiments. *p≤0.05, ***p≤0.001.

Not surprisingly the combination of distinct peptide and cytokine conditions resulted in differences in cell recovery following stimulation. Figure 2A shows the number of cells recovered from each culture condition following primary and secondary stimulation of naïve OT-II cells. The input number of responders at the initiation of secondary stimulation was normalized for each condition and thus the differences in number reflect divergence in the ability of secondary effectors to proliferate and/or survive following stimulation. In general, cells grew and survived best following stimulation with the intermediate concentration of peptide, irrespective of cytokine conditions. The reduced recovery with 10^−5^M antigen stimulation may reflect an inhibitory effect of high level TCR engagement. The low peptide concentration was the poorest with regard to cell recovery. Th1 and Th17 conditions resulted in few cells, while Th2 conditions promoted greater recovery. No Th17 cells survived following tertiary stimulation with 10^−9^M peptide, while we could maintain Th1 and Th2 cells with this peptide concentration. Thus while low peptide was in general suboptimal compared to the higher peptide concentrations, it was sufficient to sustain cultures in the presence of Th1 or Th2 cytokine conditions, but not Th17 conditions.

Although in general, the intermediate peptide level resulted in the greatest T cell growth and survival, there were cytokine-specific effects within this optimal antigen level. Th17-polarizing conditions resulted in increased recovery following both primary and secondary stimulation when using the intermediate peptide condition (Fig. 2A). A similar effect was observed at the high peptide concentration. These findings may suggest that TGFβ/IL-6 provide enhanced survival or proliferative signals compared to IL-12 or IL-4. However, importantly this effect is manifest only once a critical threshold of antigen is reached as cells could not be carried past secondary stimulation with low peptide and Th17 conditions. The increased recovery of cells following stimulation with the high or intermediate peptide dose together with Th17 differentiation conditions is consistent with studies using human CD4^+^ T cells where Th17 cells were found to be long lived cells compared to Th1 cells, which did not propagate as efficiently [31].

### Limiting Peptide Promotes Proliferation of a Restricted Population of Cells

The above data show that stimulation with limiting peptide (10^−9^M) resulted in reduced recovery compared to higher peptide concentrations. Nonetheless, Th1 and Th2 conditions did allow cells to be carried through multiple stimulations. A number of possibilities could account for the reduced recovery following stimulation with low peptide. One possibility was selective activation of a small subset of cells resulting in a decreased number of cells at d7 of culture. Alternatively, a large percentage of cells could be activated, but undergo minimal division or could divide efficiently at early times, but fail to survive over the course of the seven day culture period. To discriminate among these possibilities, we assessed division in CFSE-labeled OT-II cells isolated from naïve mice following primary stimulation. Not surprisingly, the high or intermediate peptide concentration promoted robust division with all cytokine conditions at 3d post-stimulation (Fig. 2B). Interestingly, cell recovery at d7 following primary stimulation did not fully reflect early proliferation, as Th17 cultures showed the highest recovery while exhibiting a similar division profile at 3 days post primary stimulation. Thus, differences in late proliferation or survival may be responsible for the increased recovery under Th17 conditions.

With regard to the low peptide dose, there was limited proliferation regardless of the cytokine conditions present. This proliferation was, however, dependent on the presence of peptide (compare to unstimulated OT-II cells). This was supported by our finding that survival to d7 of culture for Th1 and Th2 cells was dependent on antigen as incubation with cytokine alone was insufficient to promote recovery of live cells at this timepoint (data not shown). Together, these data suggest a minor population of cells undergo division in the presence of limiting peptide. Interestingly, the early division of cells in Th17 cultures generated in the presence of 10^−9^M peptide was not reduced compared to Th1 cultures. Thus, the failure to maintain cultures following stimulation with 10^−9^M peptide and Th17 differentiating cytokines suggest survival defect when cells were activated under these conditions.

The activation of a restricted subset of cells following culture with low peptide, together with the low cell recovery, would suggest that a significant percentage of cells in the starting population undergo death following primary stimulation. To assess this possibility we initiated cultures with naïve OT-II cells using Th1, Th2, or Th17 conditions and high, intermediate, or low peptide. At 72h following stimulation, death was assessed by staining with 7-AAD. The data in figure 2C show a significantly increased percentage of cells that were 7-AAD^+^ in cultures stimulated with 10^−9^M peptide concentration. Th2 inducing cytokines resulted in the lowest death, consistent with the increased recovery compared to Th1 and Th17 conditions (Fig. 2A).

### Early Activation Markers are Differentially Expressed by responding Cells Depending on the Combination of Cytokine and pMHC Present during Activation

**Figure 3:**
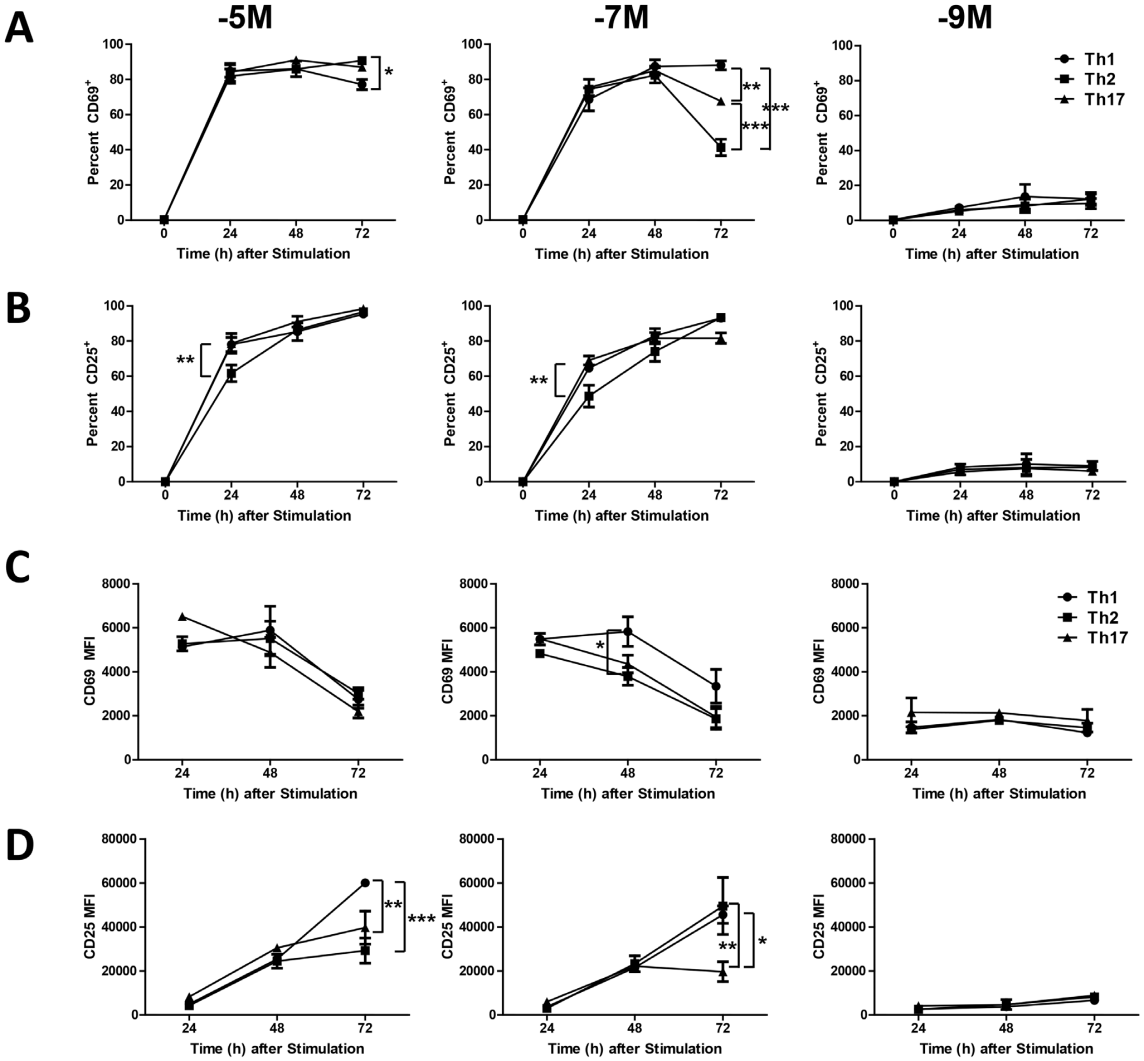
CD25 and CD69 are differentially regulated among the lines generated with high, intermediate, or low dose peptide. OT-II cells were stimulated with the high, intermediate or low concentration of peptide in the presence of Th1, Th2 or Th17 differentiating conditions and CD45.1^+^ splenocyte stimulators. At 0, 24, 48 and 72 hours, the expression of CD25 and CD69 was determined on CD4^+^CD45.2^+^ (OT-II cells). Cells were assessed for marker positivity (A and B) and the level of marker expression (C and D). Data shown are the average of 3 (A and B) or 2 (B and D) independent experiments. One experiment utilized different fluorophores for the detection of CD25 and CD69 thereby precluding averaging in C and D; however, the patterns of expression were similar. * p≤0.05, **p≤0.01, ***p≤0.001.

We next determined how the combined effect of cytokine and antigen level impacted the early activation events. We postulated that this analysis could provide insights into the differences in low dose antigen stimulated survival as well as the fitness of cultures generated under various cytokine/antigen conditions. As a measure of activation, CD69 and CD25 expression were assessed at 0, 24, 48 and 72h following stimulation. The data in figure 3A (CD69) and 3B (CD25) show that stimulation with the high or intermediate peptide concentration induced upregulation of CD25 and CD69 in a large percentage of cells. Only a small proportion of cells expressed these markers following stimulation with the low peptide concentration; however, it was increased compared to baseline (Fig. 3A and B). This increase was dependent on peptide stimulation as culture with cytokine in the absence of peptide did not result in upregulation of CD25 or CD69 above that detected at baseline, when CD25^+^ or CD69^+^ cells were virtually undetectable (data not shown). The expression of these markers on a limited number of OT-II cells following stimulation with low peptide is consistent with the reduced proliferation observed under these conditions.

No difference in the kinetics (peak expression was observed with all conditions by 24h) or maximum level of CD69 was observed among cultures stimulated with the high or intermediate peptide concentrations (Fig. 3A). There was, however, a difference in upregulation of CD25. Th2 conditions together with high or intermediate concentrations of peptide resulted in a significant reduction in the percentage of cells that upregulated CD25 at 24h compared to Th1 or Th17 cultures (Fig. 3B). However, by 48 hours all three lines were similar with regard to the percentage of cells that expressed CD69 and CD25 (Fig. 3A and B).

We also observed differences in the retention of CD69 and CD25 expression under the various conditions. With regard to CD69 expression, in general high peptide resulted in sustained CD69 positivity regardless of cytokine milieu (Fig. 3A), although there was a modest decrease in Th1 cultures. Following stimulation with the intermediate peptide dose, the percentage of CD69^+^ cells was also maintained through 72h in the Th1 conditions. However, in contrast Th17 cells decreased modestly and Th2 cells dropped to less than half of the 48h value (Fig. 3A). Thus, the cytokine environment is regulating the sustained presence of CD69. When the level of expression on positive cells was evaluated, we observed relatively similar amounts of CD69 across timepoints and culture conditions, with only the 48 hour intermediate peptide condition exhibiting a selective increase in CD69 levels on Th1 cells (Fig. 3C).

For CD25, although the percentage of cells that expressed the molecule at 72h was not substantially different, there were differences in the level of CD25 (Fig. 3D). Th1 cells generated using the high peptide concentration continued to increase CD25 between 48 and 72h and both Th1 and Th2 cells continued to increase expression following stimulation with the intermediate dose (Fig. 3D). These studies reveal that the Th1 conditions were in general, most effective at promoting maximal high level CD25 expression, with Th2 conditions second, followed by Th17 conditions which resulted in low CD25 at 72h at both high and intermediate peptide levels. Stimulation with the low peptide concentration was a poor inducer of CD25 and CD69, regardless of the cytokine conditions utilized. Together, these data show that induction, maintenance, and level of CD69 and CD25 are regulated by the combined effects of peptide and cytokines.

### The Avidity Setpoint is a Reflection of the Combined Effects of Cytokine and Antigen Concentration

Our previous studies with CD8^+^ T cells have clearly established the regulatory role of peptide in determining the peptide sensitivity, i.e. functional avidity, of effector cells [21], [22], [32], [33]. Thus we determined how peptide level impacted the functional avidity of the OT-II cells and the extent to which cytokine signals impacted peptide mediated regulation. On d7 post routine stimulation, the established cell lines were cultured with titrated concentrations of OVA_323–339_ peptide and cytokine production was measured by ICCS. The EC_50_ for cells grown under each condition was determined by designating the maximal percentage of IFNγ-producing cells as 100%. The cytokine production at each stimulating peptide concentration was then calculated based on this number. The EC_50_ value was determined following nonlinear regression curve fitting.

**Figure 4:**
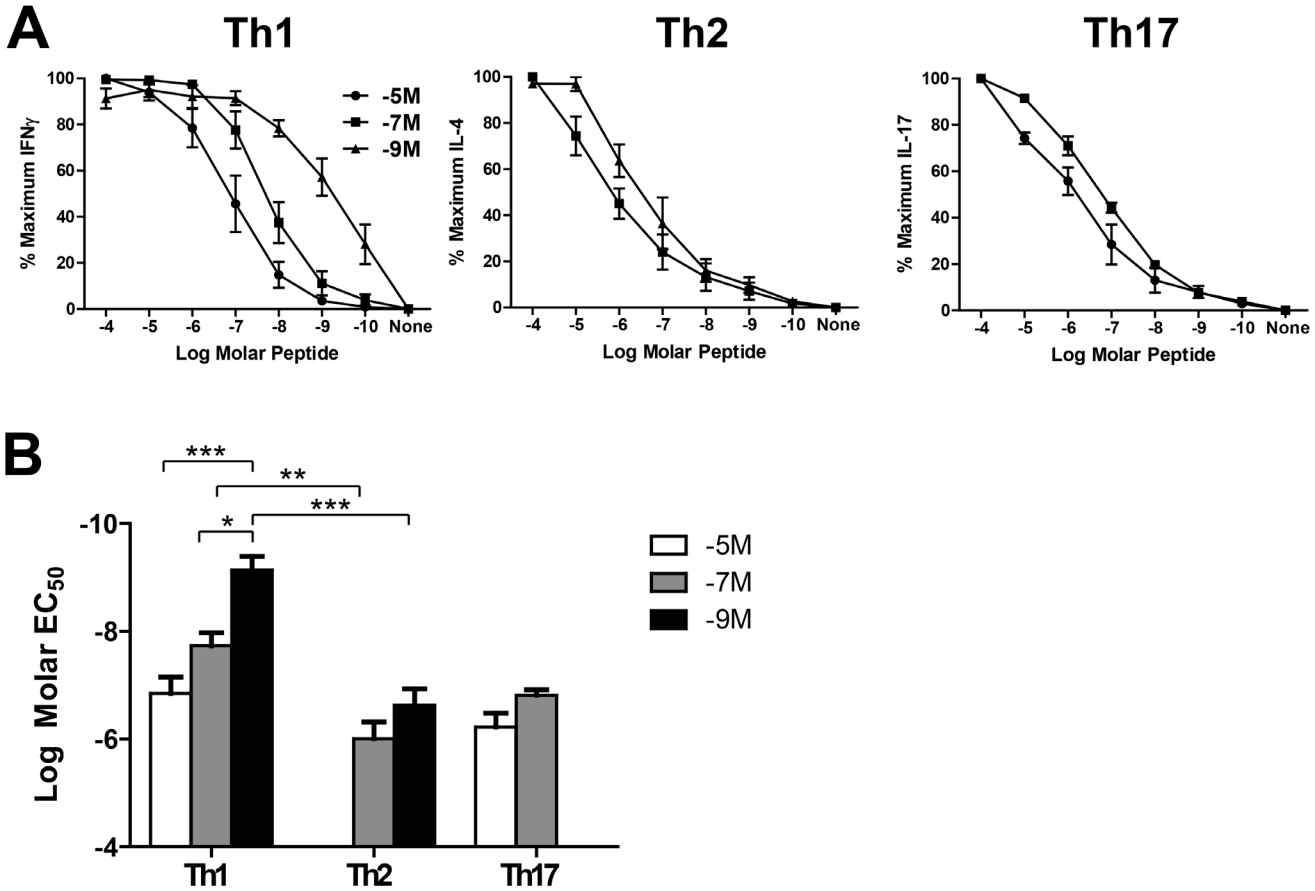
The avidity setpoint is determined by the combined effect of cytokine and pMHC level. The EC_50_ for each line was determined by stimulation with titrated concentrations of peptide. Peptide dose response curves are shown in A and averaged EC_50_ values are shown in B. Data are from 3 independent experiments in all cases, except for the 10^−5^M Th1 line (n = 4). * p≤0.05, **p≤0.01, ***p≤0.001.

Th2 lines generated with high peptide were not included because of the low percentage of functional cells in these cultures made it technically challenging to appropriately determine the EC_50_. We found that there was a dose dependent decrease in avidity among lines generated with a defined cytokine environment, i.e. cultures stimulated with decreasing amounts of peptide exhibited corresponding increases in peptide sensitivity (Fig. 4). In addition to peptide-mediated differences in avidity, there were clear effects of cytokine on the avidity setpoint. Overall, for a given level of stimulatory peptide, Th1 cells had the highest avidity followed by Th17 and finally Th2 lines (Fig. 4). Together, these data demonstrate that there are both peptide-mediated and cytokine-mediated pressures that determine peptide sensitivity in CD4^+^ T cells and it is the combined action of these signals that regulates this critical property.

### Differences in the Expression of CD4, TCR, or CD44 cannot Account for the Difference in Avidity Observed among the Subsets

**Figure 5:**
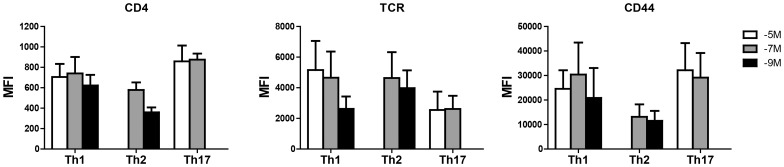
Differences in functional avidity cannot be explained by the level of CD4, TCR, or CD44. The established Th1, Th2 and Th17 cell lines were assessed for the expression of CD4, TCRβ and CD44 on d7 post routine stimulation (to circumvent any activation induced changes in level). OT-II cells were identified by CD4 and CD45.2 staining. The mean fluorescence intensity (MFI) for each marker averaged across independently generated lines is shown, n = 3 for all cases, except −5M Th1 (n = 4) and −9M Th1 (n = 2).

We next tested the hypothesis that differences in avidity in the established lines were associated with regulated expression of the TCR, CD4, or CD44. CD4 was of particular interest given previous studies showing the regulated expression of CD8 in cells undergoing avidity modulation [21], [32], [33]. While there were differences in the expression patterns of these surface molecules among the various lines, there was no correlation between their levels and avidity (Fig. 5). Thus, regulated expression of these molecules does not appear to be a mechanism utilized to control functional avidity by the CD4^+^ cells in these cultures, suggesting that regulation of intracellular signaling molecules or membrane organization may be at play.

### The Lack of Cytokine Production by Th2 Cells Generated by Stimulation with High Antigen is the Result of a Failure to Respond to Peptide Mediated TCR Activation, not an Inability to Produce Cytokine

**Figure 6:**
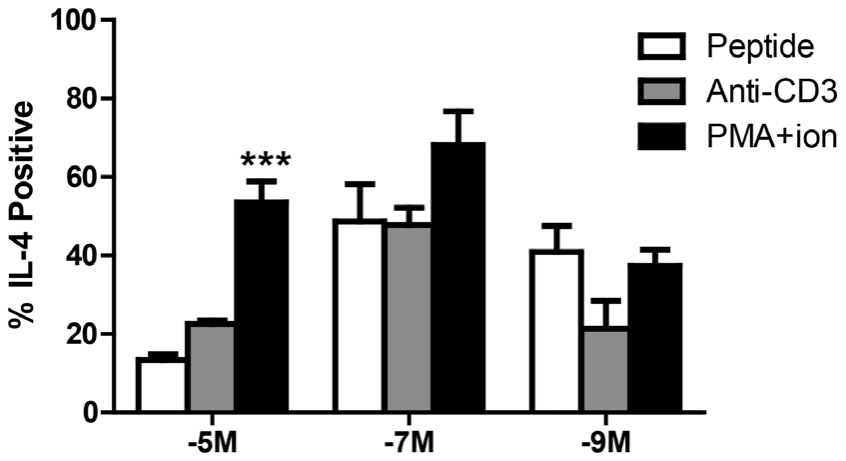
The lack of peptide responsiveness in Th2 cells generated on high dose peptide can be overcome by stimulation with PMA+ionomycin, but not anti-CD3 antibody. Established Th2 cell lines were stimulated for 5 hours in the presence of 100 µM peptide, PMA (50 ng/ml)+ionomycin (500 ng/ml), or immobilized anti-CD3 antibody (plate coated overnight with 50 µg/ml). OT-II cells were identified by CD4 and CD45.2 staining. Cytokine production was assessed by ICCS. Data shown are the average of 3 independently generated lines. *** p≤0.001.

Another aspect where the interplay of cytokine and antigen dose was evident was the regulation of functional competence following high peptide stimulation. In contrast to what was observed with Th1 and Th17 conditions, stimulation of effector cells generated in the presence of Th2 cytokines together with the high peptide concentration resulted in a very limited number of cytokine producing cells, even though cells grew and survived longterm in culture. To probe the basis for the lack of function in these cells, we determined whether this population was fundamentally unable to produce cytokine or whether the cells were selectively nonresponsive to TCR engagement of pMHC. Cells were stimulated with PMA plus ionomycin (PMA/Ion) or anti-CD3 antibody. The former bypasses TCR proximal steps in activation whereas the latter provides very high strength TCR engagement. Incubation with PMA/Ion resulted in significantly increased IL-4 production in the Th2 lines that were generated on 10^−5^M peptide (48% vs. 12%), while immobilized anti-CD3 antibody had no significant effect (Fig. 6). The increased production of IL-4 as a result of PMA/Ion addition was not observed in the Th2 lines stimulated with the intermediate or low concentration of peptide. These data suggest that there is a membrane proximal defect in signaling in Th2 cells generated with high peptide dose. Given that this defect is not present in Th17 or Th1 cells generated using the high peptide concentration, we conclude that cytokine is mediating the functional outcome of high level stimulation through the TCR with regard to differentiation into functional effectors.

## Discussion

The results from the studies presented here reveal three novel findings with regard to regulatory pressures that can shape CD4^+^ T cell subset differentiation and function. Using a well-established *in vitro* model of T cell differentiation, we find that the array of cytokines present during activation with high pMHC determines the functional competence of the resulting effectors following high peptide encounter. Second, the ability to successfully complete a program of survival and differentiation into functional Th1 vs. Th2 vs. Th17 cells is dictated by the amount of peptide utilized during activation. Finally, cytokine signals work together with pMHC to determine the avidity setpoint, withTh1 differentiation conditions leading to effectors with the highest avidity followed by Th17 and finally Th2 conditions.

The appropriate regulation of CD4^+^ T cell differentiation into distinct effector subsets is a critical determinant of pathogen clearance and inappropriate differentiation can result in reduced clearance or increased tissue damage (e.g. [1], [34], [35]). In addition, the dysregulated presence of certain subsets, e.g. Th17 cells, can contribute to disease states such as autoimmunity [36]. Thus, a detailed understanding of the environmental signals which regulate the differentiation program is critical. Further, the ability of effector cells to function in vivo is linked to their peptide responsiveness, i.e. avidity. Cells with insufficient avidity will be less efficacious in vivo in pathogen clearance, while in the case of autoimmune T cells, driving towards low avidity may be beneficial as it should limit their contribution to disease.

Antigen dose is well established as a regulator of avidity in CD8^+^ T cells. The inverse relationship between the level of pMHC and peptide sensitivity is evident for both the polyclonal response [12], [37] as well as peptide induced modulation of avidity in monoclonal TCR populations [21], [22], [32], [33]. While much less studied, there are data that suggest antigen dose can also impact CD4^+^ T cell avidity. Stimulation of polyclonal populations with a high versus low level of peptide antigen results in generation of cells with low versus high avidity, respectively [23], [24], although the ability of individual CD4^+^ T cells to undergo the peptide dependent avidity modulation described in CD8^+^ T cells has not been previously explored. We have utilized an experimental approach similar to that used in our studies of CD8^+^ T cell avidity to assess the ability of peptide level and cytokines to exert regulatory pressures on the differentiation and function of CD4^+^ T cells. Using this established approach, we show that, as was observed for CD8^+^ T cells, CD4^+^ T cell avidity can be actively modulated by the level of pMHC encountered. For a given cytokine environment, in vitro stimulation with increasingly lower peptide levels led to effectors with increasingly higher functional avidity.

While the increased sensitivity observed in effectors generated on limiting peptide might suggest that utilizing low antigen would be beneficial for immune responses, the lowest antigen concentration, although promoting highest avidity, was a poor inducer of CD69 and CD25 expression. The lack of CD69 upregulation suggests the reduced proliferation at limiting peptide is the result of the failure to initiate an activation program in the vast majority of cells. For those cells that did undergo activation as measured by upregulation of CD25 and CD69, the activation program appeared impaired, as these cells exhibited greatly reduced levels of these molecules on a per cell basis compared to cells stimulated with higher peptide concentrations. The reduced level of CD25 expression likely hinders the cell’s ability to respond to IL-2 present in the culture. Decreased responsiveness to IL-2 would explain the decreased proliferation and survival in cultures stimulated with limiting peptide. Overall these findings show that limiting peptide results in maximal avidity; however, this comes at the expense of high effector cell number. These findings have practical implications for the in vitro generation of effector cells that might be used for adoptive therapies.

The regulation of CD4^+^ T cell avidity is complex, as our studies show that in addition to peptide level, the cytokine environment contributes markedly to the avidity setpoint. We found that cytokine conditions that promote Th2 differentiation resulted in the lowest avidity for a given level of pMHC. Th17 conditions were intermediate and Th1 conditions promoted the highest peptide sensitivity. Previous in vitro studies of established Th1 and Th2 cells generated using the same peptide dose have reported differences in the regulation of TCR signaling [38]–[40] that may be explained at least in part by reduced association of TCR in lipid rafts in Th2 versus Th1 populations [41]. In addition, in contrast to Th1 cells, Th2 cells inefficiently form a “bulls-eye” immunological synapse, instead exhibiting multifocal immunological synapses [42]. Thus, cytokine signals appear to impact the membrane organization of effectors, though the mechanisms responsible for this phenomenon are currently unclear. These data suggest changes in membrane organization may be a contributor to the cytokine mediated control of peptide sensitivity.

How the presence of high vs. low peptide stimulation in combination with cytokine signals impacts membrane organization is unknown. If the TCR and cytokine signals are ultimately modulating peptide sensitivity through a common mechanism, then their effects may be additive or even synergistic. For example, if peptide level also impacts membrane organization, we would propose that lower peptide drives optimal organization and thus higher avidity. To explain our finding that Th2 conditions promote lower avidity compared to Th1 conditions, we would propose that the Th2 cytokines limit the membrane organization driven by limiting peptide, thus offsetting some of the high avidity promoting effects of this peptide dose. Alternatively, antigen dose and cytokines may regulate avidity via distinct mechanisms. For example, cytokine may work through effects on membrane organization whereas pMHC regulates avidity through modulation of signaling molecule expression (as one possibility). It will be important in future studies to define the extent to which these two modulating signals utilize distinct vs. overlapping mechanisms to regulate effector cell function.

In addition to the modulatory effects on avidity, the specific combination of pMHC level and cytokine signals present in the cultures determined subset-specific differentiation and survival. Our data show that the presence of IL-6 and TGFβ was sufficient to drive generation of Th17 cells only following culture in the presence of intermediate or high levels of peptide. The limiting peptide dose utilized here did not allow survival of cells past the initial stimulations. Thus, we cannot conclusively determine whether there was a failure to differentiate in addition to survive. In contrast, the presence of limiting peptide was capable of promoting differentiation and survival of Th1 and Th2 cells. One possibility is that the presence of Th17 conditions is inhibitory in the context of limiting peptide, although it seems more likely that there is an activation/survival advantage garnered from the presence of Th1 or Th2 conditions. While IL-12, IL-4, and IL-6 have all been shown to activate the Akt pathway that can result in upregulation of cell cycle proteins and anti-apoptotic mediators, e.g. bcl-2 [43]–[46], one could speculate that they differ in the efficiency with which they do so and that the contribution of strong TCR signals to growth and survival is more necessary in the context of Th17 conditions, perhaps due to an antagonizing effect of TGFβ.

We observed another potent regulation of cytokine and antigen dose in our studies. Whether stimulation with the high peptide dose promoted functional competence in cells was dependent on the distinct cytokines present in the environment. Th1 and Th17 conditions resulted in effectors that produced cytokine following activation with high peptide antigen. In contrast, Th2 conditions resulted in T cell survival and growth, but the cells were incapable of producing cytokine in response to peptide stimulation. Stimulation through the TCR via immobilized anti-CD3 was also incapable of inducing cytokine production from these cells. However, these cells could produce IL-4 in response to PMA and ionomycin. The ability of PMA/Ionomycin to induce IL-4 production in peptide nonresponsive Th2 cells generated by high dose stimulation shows that they did undergo appropriate subset differentiation. This is in agreement with our finding that these cells did not produce alternative subset cytokines (IFNγ or IL-17), nor did they express FoxP3 (data not shown). Cytokine production following stimulation with PMA/Ionomycin suggested a membrane proximal defect in TCR signaling. Previous studies have reported negative regulation of signaling in nonresponsive T cells. The mechanism responsible for the defects varies by the model [47]. Anergic cells generated by stimulation with anti-CD3 antibody exhibit decreases in Lck [48], whereas cells undergoing adaptive tolerance have defects in ZAP-70 activation/activity [49]. Cbl-b, Grail and Egr3 have all been implicated in peptide induced loss of function [50]–[52]. Thus, there are multiple mechanisms that could contribute to the functional defects observed in our model. Which of these are at play or whether a novel regulatory effect controls the lack of peptide responsiveness awaits further study.

The lack of TCR responsiveness in these Th2 cells is intriguing in light of our avidity findings. Our data show that Th2 conditions result in lowest avidity cells. In a model wherein overall increases in signal strength results in corresponding decreases in avidity, we would speculate that Th2 conditions provide increased supplementary signaling strength to that resulting from TCR engagement of pMHC compared to that provided by Th1 or Th17 conditions. In essence the inability of Th2 cells generated with high dose peptide to respond to peptide may reflect down modulation of avidity to the point of nonresponsiveness. There is evidence in CD8^+^ T cells that the presence of IL-4 can decrease peptide sensitivity [53] and thus a similar phenomenon may be operating there.

In conclusion, our studies reveal a complex interplay between antigen and cytokines in directing the differentiation and avidity setpoint in CD4^+^ T cells. In situations of high antigen exposure, the cytokine environment can determine whether functional T cells are generated, i.e. the presence of IL-4 may prevent the generation of functional cells. When antigen is limiting, the cytokine environment will determine whether functional cells can proliferate and survive, i.e. if Th17 cytokines are present low peptide may be insufficient to support a response. Finally, the cytokine environment together with antigen level shapes the peptide sensitivity of the cells, an attribute that has significant implications for in vivo function. These exciting findings support a model wherein a sophisticated interplay exists between TCR and cytokine signals that affects both differentiation and functional avidity in CD4^+^ T cells. Understanding the interplay of cytokine and TCR derived signals in regulating these properties in CD4^+^ T cells will increase our ability to therapeutically manipulate these populations.
